# Novel eco-friendly HPTLC method using dual-wavelength detection for simultaneous quantification of duloxetine and tadalafil with greenness evaluation and application in human plasma

**DOI:** 10.1038/s41598-024-73523-4

**Published:** 2024-10-13

**Authors:** Sayed M. Derayea, Al Amir S. Zaafan, Dalia M. Nagy, Mohamed Oraby, Al Amir, S. Zaafan

**Affiliations:** 1 Analytical Chemistry Department, Faculty of Pharmacy, Minia University, Minia, 61519 Egypt; 2 Department of Pharmaceutical Analytical Chemistry, Faculty of Pharmacy, Sohag University, Sohag, 82524 Egypt

**Keywords:** Duloxetine, Tadalafil, HPTLC-Densitometry, Dual-wavelength, Human plasma, Greenness evaluation, Medical research, Chemistry

## Abstract

A novel, environmentally friendly, and sensitive HPTLC method has been developed and validated for simultaneous quantification of duloxetine (DLX) and tadalafil (TDL) in their pure state, laboratory-prepared mixtures, and spiked human plasma. This method is particularly important for patients dealing with depression and sexual issues, as it allows for the measurement of these co-administered antidepressant and sexual stimulant drugs in biological fluids. The separation process employed a stationary phase of pre-coated silica gel 60 F_254_ and a mobile phase consisting of ethyl acetate, acetonitrile, and 33% ammonia (8:1:1, v/v). The optimized mobile phase resulted in well-defined bands for DLX and TDL, with Rf values of 0.3 and 0.8, respectively with dual-wavelength detection at 232 nm for DLX and 222 nm for TDL. Polynomial regression analysis revealed exceptional linearity for both drugs, with correlation coefficients of 0.9999 over concentration ranges of 10–900 ng/band for DLX and 10-1200 ng/band for TDL. The quantitation limits were 8.2 ng/band for DLX and 8.6 ng/band for TDL, while the detection limits (LOD) were 2.7 ng/band for DLX and 2.8 ng/band for TDL. The validation of this method followed the guidelines set by the International Council for Harmonization (ICH). Additionally, the suggested method’s greenness was assessed by means of four up-to-date ecological tools, namely the Eco-Scale, the National Environmental Method Index (NEMI), the Green Analytical Procedure Index (GAPI), and the Analytical Greenness metric approach (AGREE). The proposed method was also assessed using the Blue Applicability Grade Index (BAGI), a recently developed metric for assessing the practicality (blueness) of procedures.

**Supplementary Information:**

The online version contains supplementary material available at 10.1038/s41598-024-73523-4.

## Introduction

**Fig. 1:**
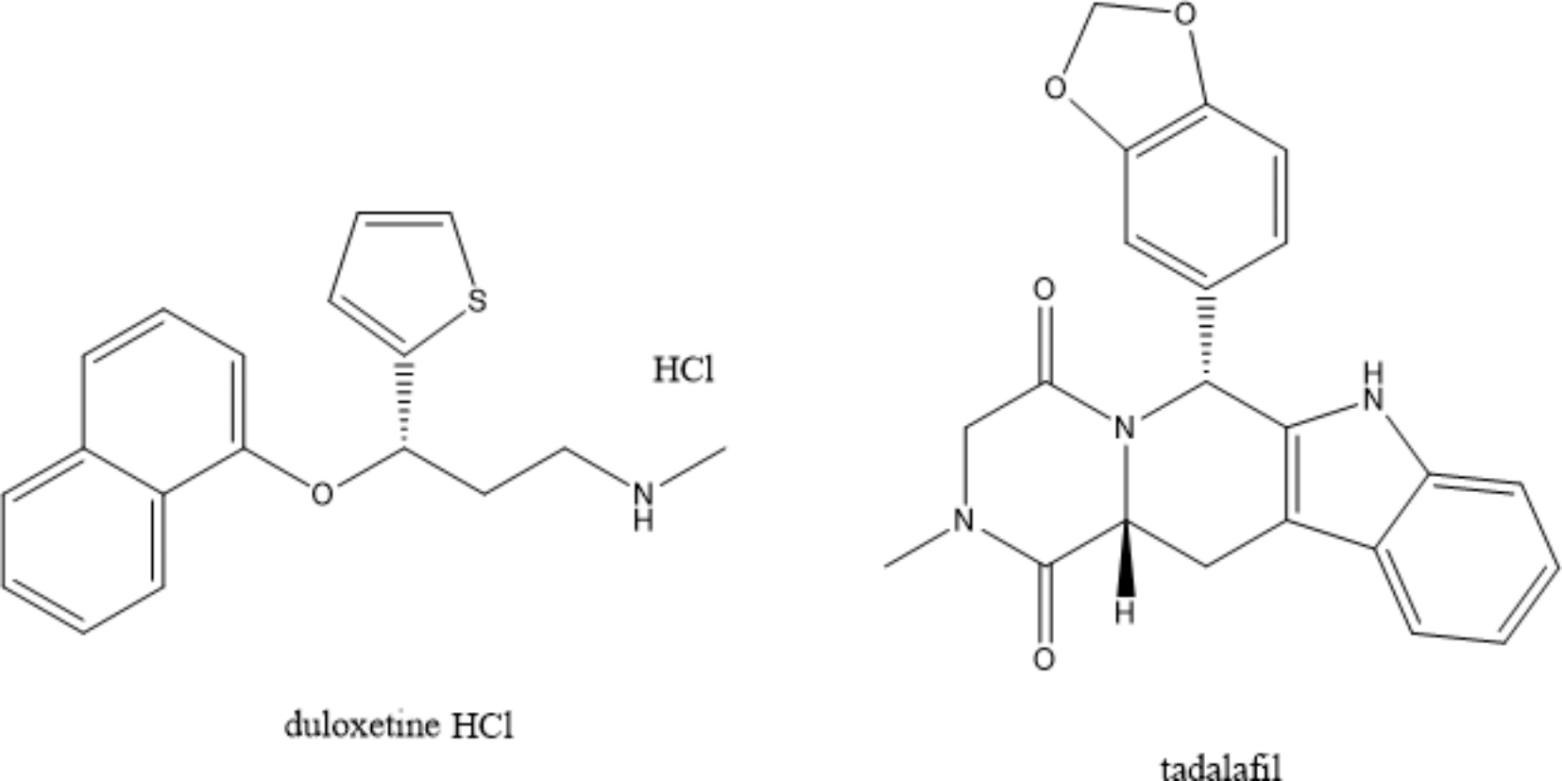
The chemical structures of the studied drugs.

Duloxetine (DLX, Fig. 1), is a chemical compound with the name of 2(+)-(S)-N-methyl-(gamma)-(1-naphthyloxy)-2 thiophen propylamine hydrochloride)^1^. It significantly inhibits serotonin and norepinephrine reuptake^2^. It is plausible to infer that DLX functions as a selective reuptake inhibitor at serotonin (5HT) and norepinephrine (NE) carriers, primarily due to its low binding affinity for opioid, histaminergic, dopaminergic, glutamate, cholinergic, and gamma-aminobutyric acid (GABA) reuptake transporters^3^. DLX has been employed to treat depression and anxiety, offering benefits such as mood enhancement, improved sleep, reduced anxiety, and increased energy, and appetite^4^. It is utilized in various countries for conditions related to NE and 5HT, including depressive disorders, diabetic neuropathy-induced pain, generalized anxiety disorder (GAD), and fibromyalgia^5^. Compared to other antidepressants, DLX has numerous benefits, including enhanced safety, increased efficacy, tolerance, and minimal undesirable effects. It also has dual inhibitory characteristics and a reduced affinity for neural receptors^6^.

Literature concerning DLX encompasses various analytical methods such as spectrophotometry^3,7–9^, spectrofluorimetry^6,10–13^, TLC^1,2,4,14,15^, electrochemical methods^16–19^, gas chromatography^20^, and HPLC^21–24^. Tadalafil (TDL) (Fig. 1) is a chemical compound bearing the name of hydro-2-methyl-6-[3,4-(methylene dioxy)phenyl] pyrazino-[1’ ,2’ :1,6] pyrido[3,4-b] indole-1,4-dione)^25^. TDL, a phosphodiesterase inhibitor, received approval in February 2003 and is utilized for managing erectile disfunction and impotence in men experiencing sexual function issues. It enhances sexual performance by augmenting blood flow in the veins of the penis^26^ and assists in controlling pulmonary arterial hypertension and erectile dysfunction^27^. The phosphodiesterase type 5 (PDE5) enzyme, which breaks down cyclic guanosine monophosphate (cGMP), is selectively inhibited by TDL^28^. This inhibition increases cGMP levels in the smooth muscle of the penile arteries, leading to muscle relaxation, improved blood flow to the corpus cavernosum, and enhanced erectile function^29^. Despite not being recognized as an official drug by pharmacopeias^30^, several methods have been published for TDL analysis, including UV spectrophotometric^25,28,31,32^, spectrofluorimetric^26,29,33,34^, TLC^27,30,35–37^, HPLC^35,38–42^, electrochemical^43–47^ and capillary electrophoresis methods^48^.

Sexual dysfunction is a recognized adverse effect of antidepressant medications, especially selective serotonin reuptake inhibitors (SSRIs) and serotonin-norepinephrine reuptake inhibitors (SNRIs)^49^. TDL can be used to treat erectile dysfunction and other undesirable sexual side effects of antidepressants. To address male reproductive issues caused by duloxetine, some physicians recommended a private prescription for phosphodiesterase-5 (PDE5) inhibitors such as TDL. Therefore, the present research focuses exclusively on the simultaneous quantification of DLX and TDL. The present study introduces a new, sensitive, selective, and environmentally friendly HPTLC method for separating and quantifying DLX and TDL in bulk, laboratory-prepared mixtures, and spiked human plasma, yielding satisfactory accuracy and selectivity results in line with the International Council for Harmonization (ICH) recommendations. This research is innovative, as no HPTLC methods for quantitatively separating DLX and TDL have been published before.

## Experimental

### Instrumentation

A TLC system from CAMAG (Muttenz, Switzerland) was utilized for the chromatographic analysis. The data was collected using Scanner 3 and processed with VisionCATS 3.1.21109.3 version software. The scanner employed a UV lamp (wavelength 254 nm, Viber Louranate 220 V 50 Hz, Marne-la-Vallee Cedex, France) as the radiation source, and it had a slit dimension of 5 × 0.2 mm, with a scanning speed of 20 mm/s. Sample application to the plate was performed using a Hamilton syringe (100 µL, Bonaduz, Switzerland), coupled with a Linomat 5 auto-sampler, with a gentle stream of nitrogen on aluminum plates coated with silica gel G 60 F_254_. The plate was developed in the ascending mode within a twin-trough chamber (standard type, 27.0 cm width × 26.5 cm height × 7.0 cm diameter, Sigma-Aldrich Co., USA).

### Chemicals, standards, and samples

DLX powder was kindly supplied as a supportive gesture by Mash Premiere pharmaceutical company (Badr City, Cairo, Egypt). TDL was generously provided by Andalous Pharma (the 6th of October city, Cairo, Egypt). HPLC grade solvents, including methanol, acetonitrile, and ethyl acetate, were procured from Merck (Darmstadt, Germany) while ammonia solution was obtained from El Nasr Pharmaceutical Chemical Co. (Cairo, Egypt).

### Standard solution preparation

An exact amount of each medication (10.0 mg) was dissolved in 8 mL of methanol with the assistance of a sonicator for 10 min. Subsequently, it was completed to 10 mL with the same solvent to create stock solutions with a concentration of 1.0 mg mL^−1^ for both drugs. The stock solutions were then diluted using the same solvent to get standard working solutions. The concentrations of these working solutions were 2, 10, 20, 60, 120, and 180 µg mL^−1^ for DLX and 2, 4, 10, 60, 120, and 240 µg mL^−1^ for TDL. To achieve the desired concentrations of 10, 50, 100, 300, 600, and 900 ng/ band for DLX and 10, 20, 50, 300, 600, and 1200 ng/ band for TDL, 5 µl of these final solutions was applied to the TLC plate.

### Procedures

#### Chromatographic conditions

Each chromatographic analysis was performed on HPTLC plates sourced from Merck, (Darmstadt, Germany). These plates were previously coated with silica gel 60 F_254_ and then divided into sections measuring 20 × 5 centimeters. To prevent the drug spot from dissolving when placed in the development chamber, each band was applied with specific parameters: it was located 1 cm away from the lower plate edge and had a width of 4 mm, with a 6 mm gap between each consecutive band. The solvent front covered a distance of 3.5 cm. The plates were dried by gently streaming nitrogen over them. This nitrogen drying method helps reduce band widening, which is particularly advantageous in HPTLC as it allows for the concurrent analysis of multiple samples in the same plate. Prior its development, the plates underwent a preliminary wash with methanol. The mobile phase employed for chromatographic separation consisted of a mixture of ethyl acetate, acetonitrile, and 33% ammonia (8:1:1 v/v). The entire volume of the mobile phase was 10 milliliters, and it took 10 min at room temperature to saturate the chamber with these mobile phase components. To prepare for analysis, the plate was dried with a hair dryer and subsequently scanned using a CAMAG TLC scanner. The scanning was performed at dual wavelengths, specifically 232 nm for DLX and 222 nm for TDL.

#### Preparation of pharmaceuticals for analysis

Ten 20 mg Tadalong^®^ tablets were crushed finely. This was used to properly weigh and transfer an amount of the tablet powder containing 50 mg of TDL into a 50 mL volumetric flask. After carefully combining the contents of ten Cymbatex^®^ 30 mg capsules, the same flask was filled with a precisely weighed amount of the mixed capsule powder corresponding to fifty milligrams of DLX. After that, methanol was used to extract the medicines, and this process involved sonicating the mixture for 30 min. Using the same solvent, the volume was then adjusted to 50 ml, and the first portion of the filtrate was then discarded. The recommended test protocol was adhered to using a mixture containing both sample solutions of the two drugs.

#### Procedure for spiked human plasma

The use of TLC for analysis of pharmaceuticals in human plasma samples is of great value^50,51^. Blood samples were taken from volunteers in good health at Misr Hospital (Sohag, Egypt). These samples were collected from the volunteers’ forearm veins and immediately placed in heparin-containing tubes. The research followed the Declaration of Helsinki Recommendations for using samples of plasma from human^52^. The “Committee on the Ethics of Scientific Research”, Faculty of Pharmacy, Sohag University, approved all studies, including the use of human plasma, and they were carried out in compliance with the Guidelines Applied to Research on Human Subjects of Sohag University. Before submitting the blood samples, each participant gave their informed written consent. To isolate the plasma, centrifugation was performed for 30 min at 4000 rpm. Subsequently, 1.0 mL of drug-free plasma was combined with 1.0 mL of the solution of the drug, which contained 20, 60, 120, and 180 µg mL^−1^ of each drug. Additionally, 2.0 mL of acetonitrile, serving as a protein precipitator, was added. The contents were thoroughly mixed, and the tube was subjected to centrifugation for 30 min at 4000 rpm. The resultant clear supernatant was gently heated on a hot plate to evaporate the organic solvent completely. The residue was then reconstituted in 1 mL of methanol and processed following the procedure outlined in the “Chromatographic conditions” section.

## Results and discussion

**Fig. 2:**
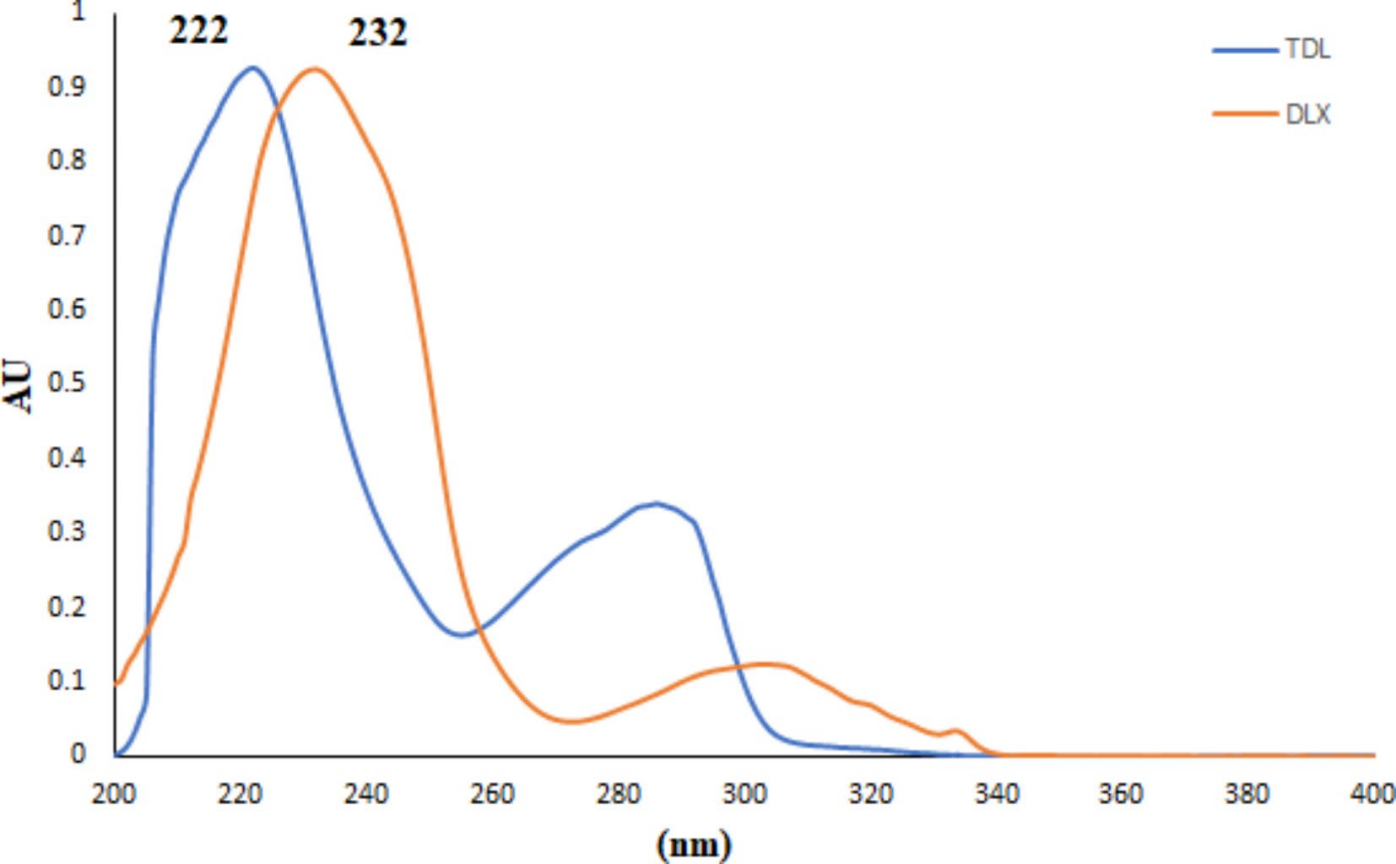
Absorption spectra of DLX and TDL.

The HPTLC method proves to be an efficient strategy for simultaneously assessing mixtures, as it offers excellent selectivity, sensitivity, and precision. Additionally, it demands minimal sample preparation and uses reduced solvent quantities, making it a cost-effective and time-saving choice. Concomitant administration of TDL with DLX provides effective treatment for sexual dysfunction resulting from DLX, there is an immediate need for a novel quantitative technique for the simultaneous assessment of the investigated medications. The currently proposed approach employs dual wavelengths and stands out for its simplicity, environmental friendliness, affordability, and ability to selectively measure both medications (Fig. 2). The method saves time, chemical resources, and effort because it facilitates the concurrent measurement of DLX and TDL in a single analysis. Due to its remarkable sensitivity and selectivity, the proposed approach allows for the measurement of both drugs in spiked human plasma, making it possible to estimate their blood levels after co-administration.

### Method development and optimization

To attain optimal resolution and separation, along with satisfactory R_f_ values and well-defined symmetrical peaks, a thorough investigation was conducted to understand the impact of various parameters on the method. This included adjustments to the mobile phase composition, the duration of saturation, the scanning wavelengths, and other factors that influenced the proposed TLC-densitometric technique.

#### Mobile phase composition

**Fig. 3:**
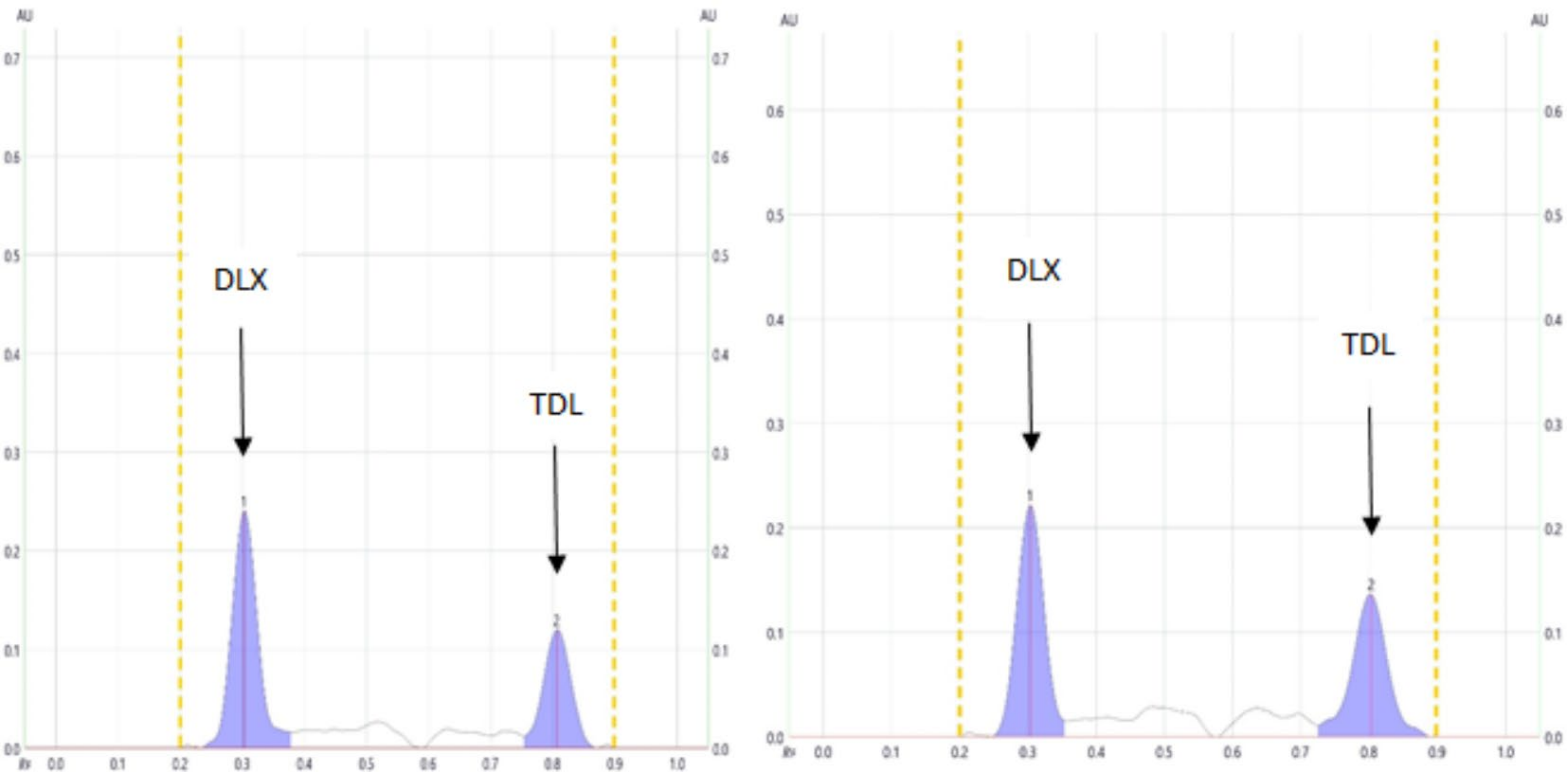
2D HPTLC densitogram of the mixture containing (300 ng/band) of DLX and (300 ng/band) of TDL scanned at dual wavelength (232 nm for DLX and 222 nm for TDL).

Utilizing the HPTLC-densitometry analysis, several mobile phase combinations were employed to investigate the separation of the binary mixture of DLX and TDL. The goal was to achieve this separation by altering the proportions of organic solvents (Table 1). It can be seen the adjustments made to the mobile phase composition after several experiments. For instance, when Methanol and acetone were mixed in an 8: 2 v/v ratio, TDL exhibited a high R_f_ value and was closely located to the front line. On the other hand, when Methanol and acetone were combined in a 2:8 v/v ratio, DLX was closely positioned to the baseline with some tailing. Another combination that was attempted was methylene chloride and methanol in a 9:1 v/v ratio, which allowed for the separation of both medications, however, there was some tailing. Furthermore, when n-hexane and ethyl acetate were mixed in a 2:8 v/v ratio, DLX remained at the baseline, while a combination of n-hexane and methanol in an 8:2 v/v ratio caused both DLX and TDL to stay at the baseline. An attempt was also made using a combination of acetone, ethanol, and 33% ammonia in an 8:1:1 v/v/v ratio, which resulted in TDL being located at the solvent front line. The best separation and resolution of the obtained peaks for the DLX and TDL combination was achieved using a developing system comprising ethyl acetate, acetonitrile, and 33% ammonia in an 8:1:1 v/v/v ratio. This resulted in an R_f_ value of 0.3 for DLX and 0.8 for TDL (Fig. 3).

**Table 1:** Different composition of mobile phases and the corresponding R_F_ for the separation of DLX ‒ TDL binary mixture.

Mobile phase	DLX *R*_F_	TDL *R*_F_
Methanol-acetone (8:2, V/V)	0.15	0.9
Methanol-acetone (2:8, V/V)	0.1 with tailing	0.9
Methylene chloride-methanol (9:1, V/V)	0.2 with tailing	0.7
n-Hexane-methanol (8:2, V/V)	At baseline	At baseline
n-Hexane-ethyl acetate (2:8, V/V)	At baseline	0.3
n-Hexane-ethyl acetate (8:2, V/V)	At baseline	At baseline
Acetone-ethanol (8:2, V/V)	0.2 with tailing	0.8
Toluene-methanol (7:3, V/V)	0.3 with tailing	0.65
Ethyl acetate-acetonitrile (8:2, V/V)	At baseline	0.6
Methanol-acetone-ammonia (8:2:0.05, V/V/V)	0.3	0.9
Acetone-methanol-ammonia (8:1:1, V/V/V)	0.8	At solvent front
Acetone-ethanol-ammonia (8:1:1, V/V/V)	0.85	At solvent front
Ethyl acetate-toluene-methanol (7:2:1, V/V/V)	At baseline	0.6
Ethyl acetate-acetonitrile-33% ammonia (8:1:1, V/V/V)	0.3	0.8

#### Saturation time

To ensure effective chromatographic separation, it was crucial for the mobile phase to thoroughly saturate the development chamber. Experimental attempts were made to achieve this by saturating the development jar with the mobile phase for different durations, ranging from 10 to 30 min. It was concluded that a 10-minute saturation period was satisfactory in achieving a suitable separation.

#### Scanning wavelength

Before scanning in the ultraviolet (UV) range between 200 and 400 nm to identify the best wavelength for detecting both DLX and TDL, a plate, containing both drugs, was prepared. Figure 2 illustrates the UV spectra for the recommended medications. The most effective scanning wavelengths for DLX and TDL, offering the highest sensitivity and selectivity, had been recognized to be 232 nm and 222 nm, respectively. These peaks were distinguishable, had symmetrical shapes, sharp contours, and were free from any noise (Fig. 3).

#### Slit dimensions of scanning light beam

By adjusting the size of the aperture through which the light beam passes during the scanning process, it becomes feasible to ensure the comprehensive coverage of all band dimensions. Additionally, this helps in preventing interference from neighboring bands. Experimentation with different aperture sizes has been conducted, revealing that the optimal aperture size for achieving the highest sensitivity is 5 × 0.2.

### Method validation

In line with the guidelines set forth by ICH, the recommended HPTLC technique underwent validation^53^, This validation encompassed assessments of its linearity range, detection (LOD), and quantitation limits (LOQ), as well as evaluations of accuracy, precision, and robustness. The expression of % recoveries was employed to elucidate the results for all these parameters.

#### Linearity

**Fig. 4:**
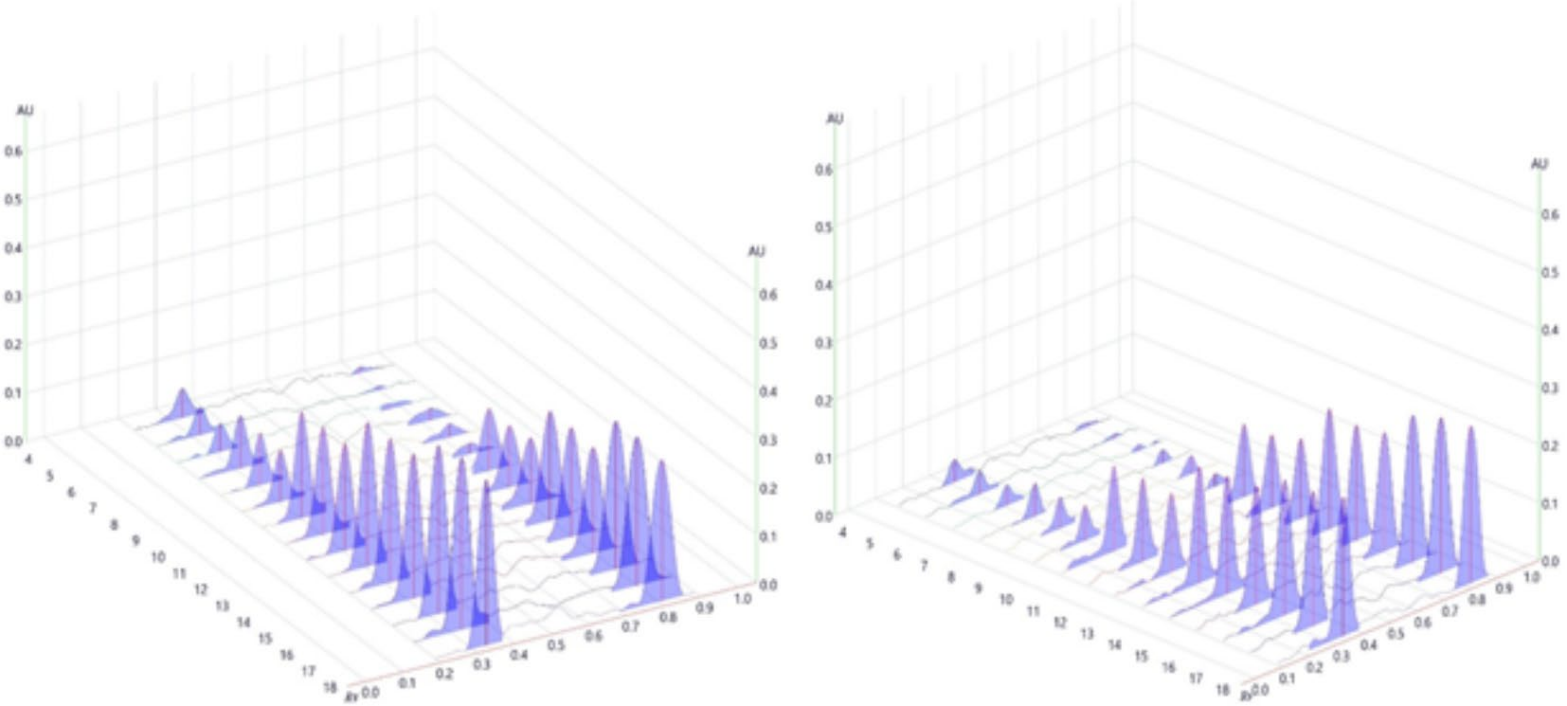
Three-dimensional HPTLC densitogram showing recorded intensities against Rf values (10–900 ng/band) for DLX and (10–1200 ng/band) for TDL, scanned at dual wavelength (232 nm for DLX and 222 nm for TDL).

The specified method for conducting the analysis was applied to assess multiple drug solutions with varying concentrations. After the plates were developed and scanned at wavelengths of 232 and 222 nm, the values for peak areas were determined and then plotted against drug concentrations measured in ng/ spot, to create calibration curves. Each concentration underwent five measurements. It became evident that a residual graph demonstrated a superior fit to the data when using a second-order polynomial, as opposed to a linear regression equation, with notably higher determination and correlation coefficients. The data were fitted to the polynomial equation: y = ax^2^ + bx + c. The relationship was directly proportional within concentration ranges of 10–900 ng/ spot for DLX and 10–1200 ng/ spot for TDL (Fig. 4). The calculated correlation (*r*) and determination coefficients (*r*^2^) were exceptionally high, with *r* = 0.9999 and *r*^2^ = 0.9999 for the quadratic polynomial fit, signifying a substantial correlation between the peak areas obtained and the concentrations examined. Additional statistical parameters for the second-order polynomial regression equation for both medications are demonstrated in Table 2.

**Table 2:** Statistical data of some analytical parameters of the proposed method.

Parameters	Tadalafil	Duloxetine
Linear range (ng/spot)	10-1200	10–900
Coefficient x ± SD	1.79E-05 ± 8.76E-08	4.24E-05 ± 5.81E-07
Coefficient x2 ± SD	−5.414E-09 ± 7.237E-11	−2.1295E-08 ± 6.355E-10
Intercept ± SD	0.0002 ± 1.544E-05	0.0008 ± 8.27E-05
Correlation coefficient (r)	0.9999	0.9999
Determination coefficient (r^2^)	0.9999	0.9999
Number of determinations	5	5
Limit of detection (ng/spot)	2.85	2.71
Limit of quantitation (ng/spot)	8.62	8.22

#### Detection and quantitation limits

For the purpose of the assessment of the method’s sensitivity, the LOQ and LOD were determined. Due to the notably low LOD and LOQ values for DLX and TDL, it was evident that the recommended approach is exceptionally sensitive, as illustrated in Table 2. The LOD and LOQ values were computed by utilizing the standard deviation of the intercept (σ) and the coefficient of the X variable in the polynomial regression equation (S). The following formulas were employed;

LOD = 3σ/S.

LOQ = 10σ/S.

Specifically, the calculated LOD values were 2.7 ng/ band for DLX and 2.8 ng/ band for TDL, while the calculated LOQ values were 8.2 ng/ band for DLX and 8.6 ng/ band for TDL (Table 2).

#### Accuracy

The study assessed the %recovery of both medications at three different concentration points, covering the low, intermediate, and high levels of the calibration curve (100, 300, and 900 ng/ band for both medications). This assessment was conducted by the recommended procedure, involving three separate measurements for each concentration level. The results indicated that the estimated percentage recovery values were nearly 100%, and the standard deviation values were minimal, demonstrating the exceptional accuracy of the suggested approach (Table 3).

**Table 3:** Evaluation of accuracy of the analytical HPTLC procedure for determination of the investigated drugs.

Drug	Conc. (ng/spot)	Amount found (ng/spot)	% Recovery ^a^ ± SD
Tadalafil	100	101.67	101.67 ± 2.10
	300	301.05	100.35 ± 1.33
	900	881.64	97.96 ± 1.28
Duloxetine	100	99.72	99.72 ± 2.24
	300	293.04	97.68 ± 2.02
	900	892.17	99.13 ± 1.15

^**a**^Mean of three determinations, SD, standard deviation.

#### Precision

The precision of the suggested method was evaluated at two levels: intra-day (repeatability) and inter-day (intermediate). To evaluate repeatability, three different concentrations within the linear range (100, 300, and 900 ng/ band for both medications) were analyzed three times in a single day using the recommended procedure. For assessing intermediate precision, the same analysis was performed three times over three consecutive days. All measurements were conducted in triplicates. The results indicate that the proposed method exhibits a high level of precision at both intra-day and inter-day levels. This is evident from the calculated % recovery values, which were very close to 100%, and the % relative standard deviation (%RSD) values, which were quite low, as demonstrated in Table 4.

**Table 4:** Evaluation of intra-day and inter-day precisions for the determination of the investigated drugs with the proposed method.

Drug	Conc. (ng/spot)	Intra-day precision % recovery ± SD^a^	%RSD	Inter-day precision % recovery ± SD^a^	%RSD
Tadalafil	100	102.68 ± 2.17	2.11	100.69 ± 2.33	2.31
	300	100.82 ± 0.50	0.50	99.56 ± 2.28	2.29
	900	99.04 ± 0.79	0.80	99.14 ± 1.99	2.01
Duloxetine	100	101.97 ± 1.89	1.85	102.10 ± 2.22	2.18
	300	97.55 ± 2.16	2.21	97.05 ± 2.14	2.20
	900	99.14 ± 1.14	1.15	99.37 ± 1.49	1.50

^**a**^ Mean of three determinations, SD, standard deviation, %RSD, relative standard deviation.

#### Robustness

Deliberate, minor changes were made to the variables in the HPTLC procedure to evaluate the resilience of the suggested methodology. The effects of these modifications on the outcomes were investigated. The alterations involved slight changes to the mobile phase composition, saturation time, and detection wavelength. The findings indicated that the alterations made to these parameters had no noteworthy influence on the analysis of both medications, as illustrated in Table 5. This confirmed that the suggested approach is both sturdy and dependable.

**Table 5:** Evaluation of the robustness of the proposed HPTLC method.

Normal condition	Changed condition	% recovery ± SD^a^ TDL 600 (ng/spot)	% recovery ± SD^a^ DLX 600 (ng/spot)
Mobile phase composition ethyl acetate-acetonitrile-ammonia (8:1:1, V/V)	(7.8:1.2:1, V/V)	100.06 ± 0.51	100.55 ± 2.29
	(8.2:0.8:1, V/V)	100.48 ± 1.75	100.51 ± 2.49
	(7.8:1:1.2, V/V)	102.73 ± 1.26	100.68 ± 1.72
	(8:0.8:1.2, V/V)	99.51 ± 0.97	100.21 ± 1.59
*Wavelength*			
TDL 222 nm	220 nm	97.83 ± 1.42	
	224 nm	96.89 ± 2.04	
DLX 232 nm	230 nm		97.44 ± 1.18
	234 nm		98.83 ± 1.76
Chamber saturation time 10 min	8 min	98.33 ± 0.98	99.81 ± 0.57
	12 min	102.32 ± 1.98	100.56 ± 0.96

^a^Mean of three determination, SD, standard deviation.

#### Specificity

The complete isolation of DLX and TDL was successfully achieved, as evidenced by the HPTLC chromatogram (Fig. 3). This outcome confirms the precision of the suggested approach. Furthermore, the lack of peaks at the R_f_ of the medications and the method’s effective utilization in the synthetic mixture illustrate the absence of any interference from excipients (Table 6).

**Table 6:** Comparison between the results of the estimation of DLX and TDL in their pharmaceutical preparation using the proposed and reported methods.

Parameter	Proposed method		Reported method	
	Duloxetine	Tadalafil	Duloxetine	Tadalafil
Number of measurements	5	5	5	5
Mean % recovery^a^	100.42	101.32	100.86	99.59
SD	0.65	1.63	0.81	2.14
t-test^b^ (2.306)	0.95	1.43		
F-value^b^ (6.388)	1.31	1.67		

^a^Mean of five measurements, SD, standard deviation.

^b^Tabulated value at 95% confidence limit, F = 6.388 and t = 2.306.

#### Stability and recovery

The plasma samples that were spiked with TDL and DLX underwent three freeze-thaw cycles, three days of short-term stability at -20˚C, and also six hours of bench stability at room temperature. After exposing the samples to these different stability studies, triplicate of the spiked human plasma samples was examined. The stability samples’ mean concentrations were contrasted with those of the freshly prepared samples. and the results are expressed in terms of % recovery. Results of the study are summarized in the table presented in S1.

### Application of the suggested method

#### Application to pharmaceutical preparations

Using the recommended HPTLC method, the laboratory-made solutions of Cymbalta^®^ 30 mg and Tadalong^®^ 20 tablets, the commercially available local market products were successfully investigated. The results were compared with those obtained using established methods^8^ for DLX and^28^ for TDL (Table 6). For these combined components, statistical tests such as the t- and F-tests at 95% confidence levels showed that the findings gathered using the suggested approach and the published methodologies did not differ significantly. These findings demonstrate the suitability of the approach for the investigated drugs, with satisfactory recovery, and no interference from co-administered medications or commonly encountered excipients. The suggested method is characterized by precision, accuracy, selectivity, and sensitivity. It is well-suited for application in quality-control laboratories and the investigation of the studied medications in their various pharmaceutical formulations.

#### Application to spiked human plasma

**Fig. 5:**
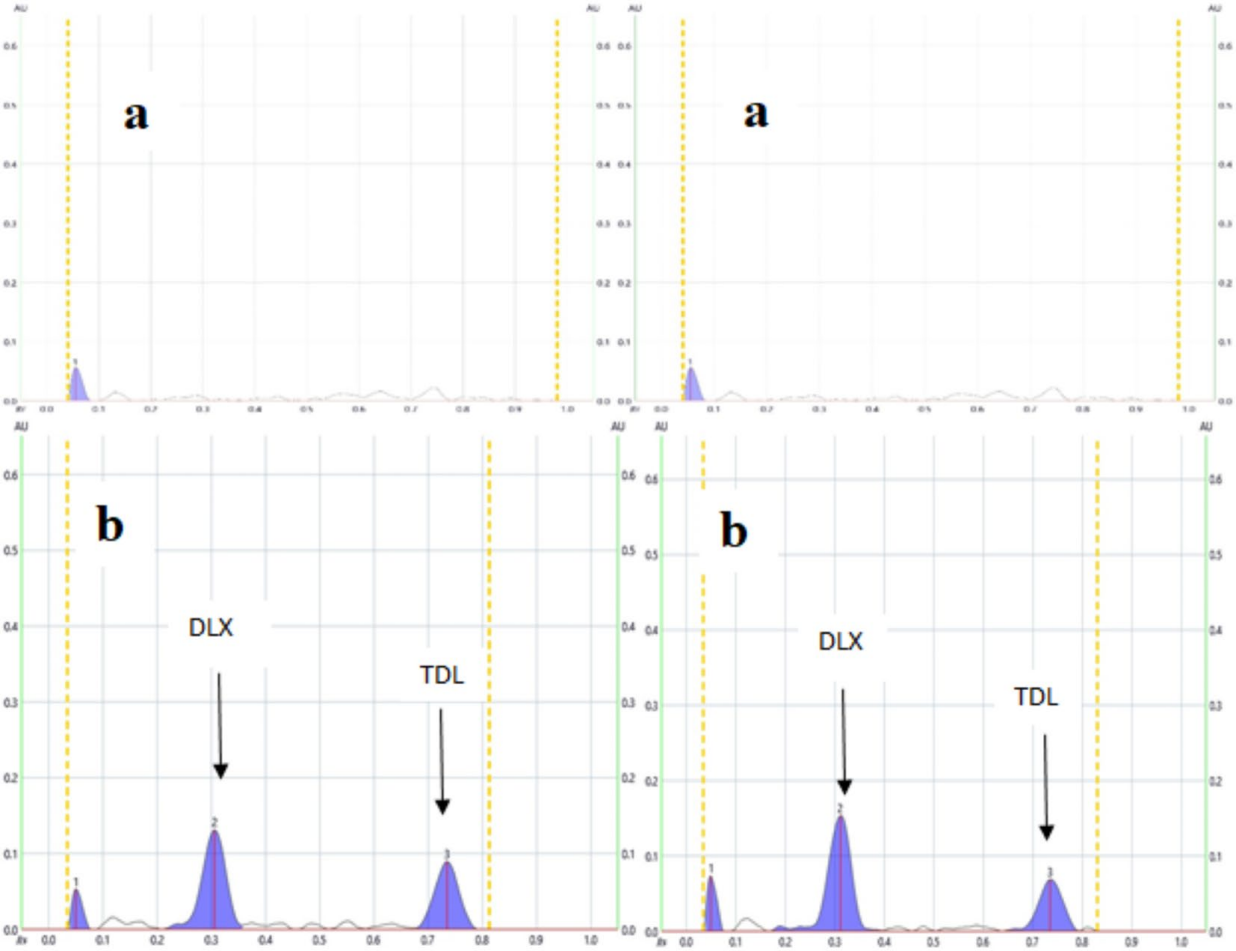
2D HPTLC densitogram of plasma sample (**a)** before and (**b**) after spiking with (300 ng/band) of DLX and (300 ng/band) of TDL measured at dual wavelength (232 nm for DLX and 222 nm for TDL).

The maximum plasma level (c _max_) of DLX and TDL are 110 and 378 ng/mL,^5,54^ respectively, while their limits of quantitation using the present method are 8.2 and 8.6 ng/ band for DLX and TDL, respectively. Thus, the examination of the DLX-TDL mixture in human plasma became feasible due to the highly sensitive HPTLC method employed in this study. To prepare the samples, a simple process of protein precipitation using acetonitrile and subsequent centrifugation was performed. These plasma samples were then enriched with four distinct concentrations of the drug mixture and analyzed using the recommended protocol. The method blank analysis was conducted on a portion of plasma that hadn’t been spiked with any drugs (Fig. 5a). Notably, the analyzed samples exhibited effective separation of the two medications on the TLC plate, with clear identification (Fig. 5b). In this analysis, the plasma components displayed faint but well-defined peaks from both medications, indicating minimal interference with the results (Table 7). As a result, it was determined that the proposed methodology is suitable for measuring these examined medications in spiked human plasma, as evidenced by the high %recoveries obtained. This method also offers the advantage of determining the blood levels of these researched medications following co-administration. It’s worth noting that the study opted to work with in vitro human plasma samples combined with the medications because obtaining blood samples from individuals undergoing treatment with these drugs proved to be challenging. The calculated standard deviations fell within the range of 0.62 to 3.42 for DLX and 0.78 to 3.51 for TDL. These values reflect the minimal variability attributed to the matrix’s influence on the quantification of DLX and TDL in human plasma. It should be revealed that nearly every previously published technique was used to determine either TDL or DLX alone or in combination with other medications. Therefore, the current work’s significance arises from its ability to measure both medications concurrently. For concurrent estimation, a new spectrofluorimetric approach was presented lately. The suggested approach uses fewer chemicals and can analyze many samples in a single run, making it faster even if the published method has higher sensitivity. In the supplementary data (S2), a comparison of our approach with the recently released publication is provided.

**Table 7:** The application of the developed method to measure the drug in spiked human plasma.

Drug	Conc. (ng/spot)	Amount found (ng/spot)	% Recovery^a^ ± SD
Tadalafil	100	102.33	102.33 ± 0.78
	300	289.38	96.46 ± 1.77
	600	598.32	99.72 ± 1.55
	900	925.56	98.16 ± 1.84
Duloxetine	100	101.04	101.18 ± 1.94
	300	279.45	93.15 ± 0.62
	600	553.02	92.17 ± 1.84
	900	869.22	96.58 ± 1.44

^a^Mean of three determination, SD, standard deviation.

### Method greenness evaluation

**Fig. 6:**
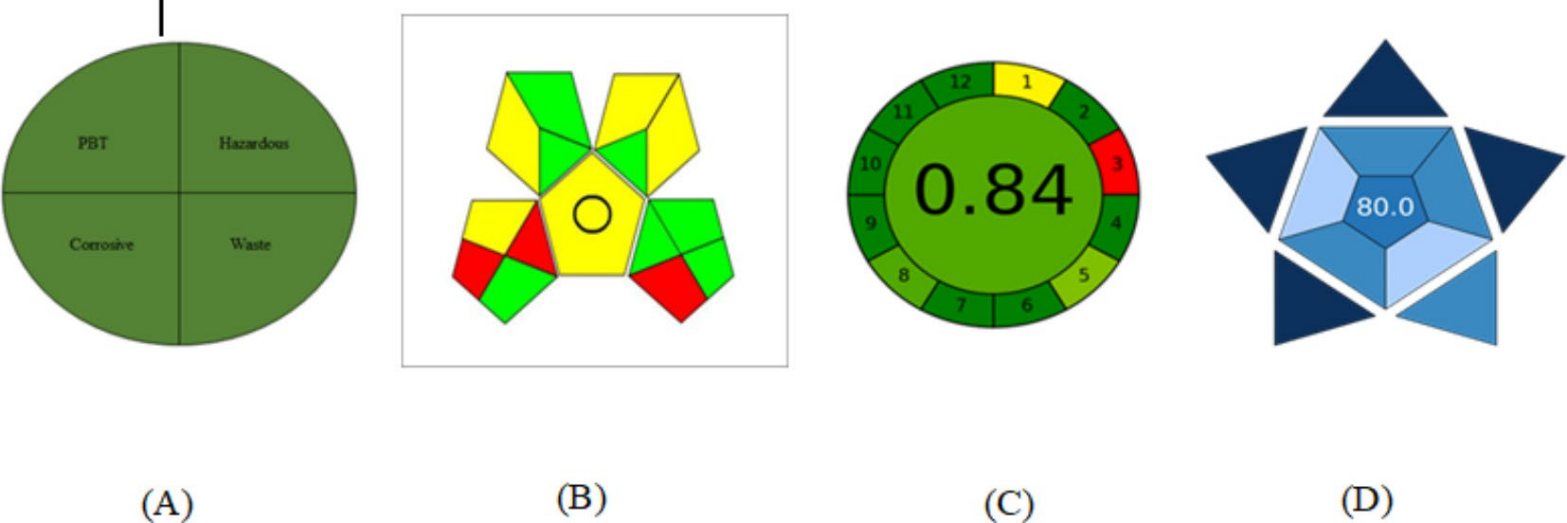
Evaluation of the proposed HPTLC method using NEMI pictograms (**A**), GAPI (**B**), AGREE (**C**), and BAGI (**D**) tools for greenness and blueness.

The concept of a “green” analysis revolves around three key attributes: the absence of waste, minimal or non-existent use of hazardous chemicals, and reduced energy consumption. To evaluate the environmental friendliness of the suggested technique, several evaluation tools are available for consideration^55,56^. In assessing the greenness of the recommended TLC densitometric approach, the analysis focused on the greenness profile, Eco-Scale methodology, the Green Analytical Procedure Index (GAPI) and the Analytical Greenness metric approach and software (AGREE). The greenness profile of the established technique was appraised by the National Environmental Method Index (NEMI)^57,58^, and the method met all the criteria for being considered a green approach (Fig. 6). This was achieved by utilizing solvents that are non-persistent, non-bio accumulative, and non-toxic (PBT). The suggested approach employed non-PBT solvents such as acetonitrile and ethyl acetate. Additionally, the pH level of the developing system was close to neutral and therefore not corrosive. The chemicals used in the method were non-hazardous, and the waste generated was minimal, amounting to less than 50 mL. Based on these findings, the suggested TLC-densitometric method was deemed environmentally friendly, meeting all four quadrants of the greenness profile. The Eco-Scale method provides a straightforward approach to assessing the ecological impact of the proposed method in quality-control laboratories. It calculates penalty points for various parameters, including the amount of chemicals used, workplace hazards, waste generation, and energy consumption. The analytical Eco-Scale score was determined using the equation (analytical Eco-Scale score = 100 - total penalty)^59,60^. It is considered excellent if it exceeds 75. In the current research, the use of non-toxic solvents, minimal waste generation, and low energy consumption contributed to a high ecological friendliness rating, with an Eco-Scale score of 84 (Table 8). The Green Analytical Procedure Index (GAPI) is an additional metric used to evaluate the greenness of the suggested approach^61,62^. GAPI evaluates various aspects of the analytical process, assigning color codes (green, yellow, and red) to indicate minimal, moderate, or significant environmental impact. In this evaluation, the GAPI pentagrams revealed that the proposed approach achieved a satisfactory level of greenness, with 7 green, 6 yellow, and 3 red fields (Fig. 6). An additional green metric was added to provide a more thorough understanding of the exceptional green aspects of the recommended strategy. The term “AGREE analytical tool” refers to this metric^63^. The twelve major GAC principles are followed in the arrangement of the twelve segments that constitute the AGREE framework. As you can see, every part in these sections has a unique color that represents its level of greening, which ranges from 0.0 (represented by the color red) to 1.0 (represented by the color green). All of these principles are evaluated thoroughly in order to determine the final score. A detailed assessment of each of these principles determines the final score. In the center of the framework, a clock-like pictogram serves as both a color and a score indicator for the overall procedure demonstrating this rating. Software that produces automatically a report and a graph could potentially be used to carry out the evaluation.

**Table 8:** Evaluation of the greenness of the proposed HPTLC method using Eco scale score method.

**Parameters**		**Penalty points**
Reagents	Ethyl acetate	4
	Acetonitrile	4
	Ammonia solution	6
Instrument		
Energy consumption	(Less than 1.5 kWh per sample)	1
Occupational hazard	(Analytical process hermitization)	0
Waste	(<1 mL)	1
Total penalty points		16
Analytical eco-scale total score		84

^**a**^If the score is greater than 75, it represents excellent green analysis. If the score is greater than 50, it represents acceptable green analysis. If the score is less than 50, it represents inadequate green analysis.

### Blueness evaluation

The Blue Applicability Grade Index (BAGI), an innovative metric tool, is provided to assess the applicability of an analytical approach^64,65^. The BAGI tool yields two separate groups of outcomes: a numerical score in the center and a graphical representation in the form of an asteroid pictogram. The evaluation’s results are represented visually by the asteroid-shaped pictogram, which is composed of numerous blue hues to indicate different compliance levels (dark blue for high, blue for moderate, light blue for low, and white for non-compliance).

The recommended method receives an overall rating of 80.0, as can be seen in the center of the pictogram. BAGI takes into consideration 10 aspects in order to provide a pictogram and a score that shows the practicality of an analytical technique (S3). An orderly blue color scale was utilized for illustrating the final rating, with different degrees of dark blue, blue, light blue, and white designating high, medium, low, and no compliance of the procedure with the specified criteria, respectively. It is recommended that the final score be higher than 60, so that the analytical method can be considered “practical”.

## Conclusion

A novel, precise, straightforward, environmentally friendly, and extremely sensitive TLC method was developed to determine DLX-TDL combinations. Using the Eco-scale, NEMI, GAPI, AGREE, and BAGI metrics, the suggested methodology was evaluated. The results unequivocally show the ecological compatibility and sustainability of the suggested approach. This method is exceptionally straightforward, rapid, selective, eliminates the need for extraction steps, and utilizes a cost-effective technique that offers exceptional accuracy and precision. Furthermore, this approach exhibits remarkable sensitivity, enabling the accurate measurement of the targeted medications in plasma without significant interference from plasma components. This is especially valuable when assessing the concentrations of co-administered sexual stimulants and antidepressants in biological fluids for individuals suffering from depression. Notably, the suggested method boasts various advantages, including easy sample application, swift analysis, the ability to process a large number of samples in each run, and least solvent usage. In addition, this proposed methodology surpasses previously reported high-HPTLC techniques in terms of sensitivity. Unlike traditional chromatographic methods that often employ solvents known for their adverse health and environmental effects, this innovative approach is environmentally conscious and can be readily applied in quality control laboratories.

## Electronic supplementary material

Below is the link to the electronic supplementary material.

Supplementary Material 1

## Data Availability

The datasets used and/or analyzed during the current study available from the corresponding author on reasonable request.
