# Supplementary material for: Novel eco-friendly HPTLC method using dual-wavelength detection for simultaneous quantification of duloxetine and tadalafil with greenness evaluation and application in human plasma
Source: Sci Rep. 2024 Oct 13;14:23907. doi: 10.1038/s41598-024-73523-4 (PMC11471823; doi:10.1038/s41598-024-73523-4)
Supplement: Supplementary file 1 — Supplementary Material 1 [file 41598_2024_73523_MOESM1_ESM.docx]

S1 (Table): Stability of duloxetine and tadalafil under various conditions at two concentrations.

| **QC Level** | **Duloxetine** | | **Tadalafil** | |
| --- | --- | --- | --- | --- |
|  | **% recovery ^a^** | **% RSD** | **% recovery** | **% RSD** |
|  | **Stability at room temperature for 6 h** | | | |
| **LQC** | 99.28 | 0.009 | 99.38 | 2.88 |
| **HQC** | 96.95 | 2.68 | 100.54 | 0.48 |
|  | **Stability at -20 for 10 days** | | | |
| **LQC** | 99.39 | 0.006 | 101.33 | 0.97 |
| **HQC** | 91.83 | 5.97 | 99.99 | 0.04 |
|  | **Freeze thaw stability** | | | |
| **LQC** | 99.39 | 0.006 | 101.09 | 5.77 |
| **HQC** | 102.53 | 4.86 | 99.65 | 0.66 |

**^a^** Mean of three determination, RSD, relative standard deviation, LQC, low quality control, HQC, high quality control.

S2 (Table): Comparison between the proposed and reported methods regarding analytical performance.

| **Linearity** | | **LOD** | | **LOQ** | | **Greenness evaluation** | **Ref.** |
| --- | --- | --- | --- | --- | --- | --- | --- |
| **DLX** | **TDL** | **DLX** | **TDL** | **DLX** | **TDL** |  |  |
| 0.1-1.5 μg mL^−1^ | 0.01-0.40 μg mL^−1^ | 0.013 μg mL^−1^ | 0.004 μg mL^−1^ | 0.040 μg mL^−1^ | 0.012 μg mL^−1^ | 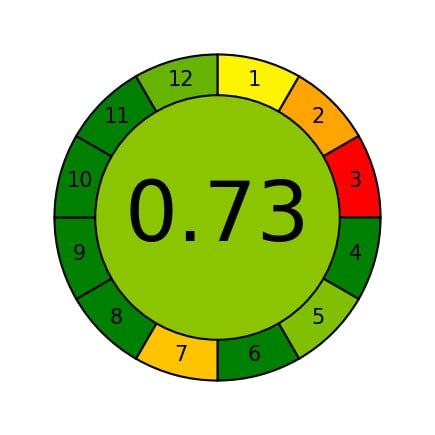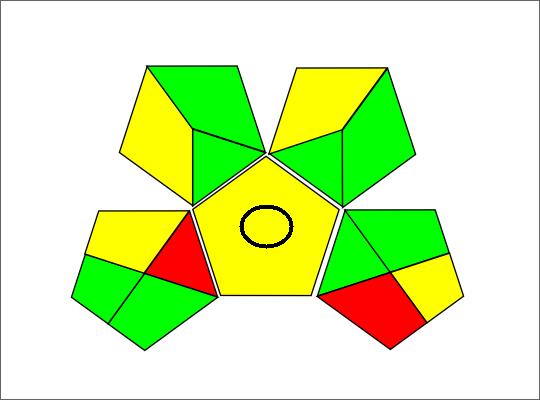 | [1] |
| 10-900 ng spot^−1^ | 10-1200 ng spot^−1^ | 2.71 ng spot^−1^ | 2.85 ng spot^−1^ | 8.22 ng spot^−1^ | 8.62 ng spot^−1^ | 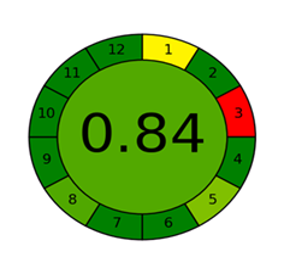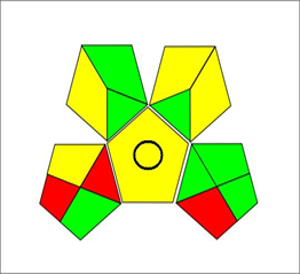 | This work |

S3 (Table): The 10 factors utilized in evaluation of the proposed method using Blue Applicability Grade Index (BAGI)

| **Parameter** | **Rating** | **Remarks** |
| --- | --- | --- |
| 1. Type of Analysis | moderately blue | Method is categorized as quantitative. |
| 1. Multi-Analyte Procedure | light blue | It determines two components. |
| 1. Analytical Technique Used | moderate blue | Densitometric apparatus which is an instrument that is readily available in most labs. |
| 1. Simultaneous Sample Preparation | light blue | The proposed technique of simultaneous preparation may be accomplished fast and without difficulty. |
| 1. Sample Preparation | moderately blue | Sample preparation is easy and affordable. |
| 1. Samples Per Hour | dark blue | An immense number of samples (Batch analysis) |
| 1. Availability of Reagents | dark blue | Ordinary, readily available chemicals are employed instead of derivative reagents. |
| 1. Preconcentration | dark blue | There is no need for preconcentration. |
| 1. Automation of Device | dark blue | It makes use of a semiautomated equipment. |
| 1. Amount of Samples | moderate blue | Sample is less than 100 µL |
